# Sigmoid Volvulus in a Pregnant Woman: A Rare Case Report of Intestinal Obstruction During Pregnancy That Requires Prompt Surgical Intervention

**DOI:** 10.1155/crog/7321070

**Published:** 2026-04-02

**Authors:** Berat Krasniqi, Arben Haliti, Mentor Sopjani

**Affiliations:** ^1^ Faculty of Medicine, University of Prishtina, Prishtina, Kosovo, uni-pr.edu; ^2^ Department of Gynecology and Obstetrics, University Clinical Center of Kosova, Prishtina, Kosovo, shskuk.org

**Keywords:** case report, gastrointestinal obstruction, pregnancy, sigmoid volvulus, surgical intervention

## Abstract

**Background and Aims:**

Sigmoid volvulus (SV) is a common cause of large‐bowel obstruction in elderly patients but represents a rare and potentially life‐threatening cause of intestinal obstruction during pregnancy. Physiological and anatomical changes in pregnancy may delay diagnosis, increasing maternal and fetal risks. This case report aims to highlight the diagnostic and therapeutic challenges of SV in pregnancy.

**Methods:**

We report the case of a 32‐year‐old pregnant woman at 27 weeks and 4 days of gestation who presented with acute abdominal pain, nausea, and constipation. Clinical evaluation, laboratory testing, abdominal ultrasound, magnetic resonance imaging (MRI), and colonoscopy were used to establish the diagnosis.

**Results:**

SV was confirmed, and endoscopic detorsion was attempted twice as first‐line management but was unsuccessful. Given persistent obstruction and symptom severity, surgical intervention was undertaken. The patient underwent sigmoid colon resection with colostomy (Hartmann procedure). Both maternal and fetal postoperative courses were favorable, and the pregnancy was successfully prolonged to term.

**Conclusion:**

Although rare, SV should be considered in pregnant women presenting with symptoms of intestinal obstruction. Early diagnosis, appropriate imaging, and a multidisciplinary approach are essential. Endoscopic detorsion remains the first‐line treatment when feasible, while timely surgical management is essential in cases of failed decompression to optimize maternal and fetal outcomes.

## 1. Introduction

Sigmoid volvulus (SV) is an uncommon cause of intestinal obstruction during pregnancy. Intestinal obstruction in pregnant women most frequently results from adhesions, hernias, and malignancies, while SV represents the most common cause of colonic obstruction in this population [1]. Although SV is a well‐recognized surgical emergency in the general population—particularly among elderly individuals [2]—its occurrence during pregnancy is rare but associated with substantial maternal and fetal morbidity when diagnosis or treatment is delayed.

The term volvulus derives from the Latin “volvere,” meaning “to twist,” and refers to axial rotation of a bowel segment around its mesenteric attachment, leading to luminal obstruction and potential compromise of mesenteric blood flow [2]. In SV, progressive bowel distension and vascular impairment may rapidly result in ischemia, necrosis, and perforation if timely intervention is not undertaken [3, 4]. Pregnancy‐related factors, including mechanical displacement of the sigmoid colon by the enlarging uterus and progesterone‐mediated reduction in gastrointestinal motility, are believed to increase susceptibility to volvulus, particularly during the second and third trimesters [5].

The clinical presentation of SV during pregnancy does not differ fundamentally from that in nonpregnant patients and typically includes abdominal pain, distension, constipation, and vomiting. However, these symptoms may overlap with common pregnancy‐related complaints, which can delay diagnosis and definitive management [6]. Diagnostic evaluation may be further complicated by concerns regarding fetal exposure to ionizing radiation, underscoring the importance of appropriate imaging strategies, including the selective use of magnetic resonance imaging (MRI) when clinically indicated [5].

Recent evidence indicates that maternal and fetal outcomes in pregnancy‐associated SV are strongly dependent on the timeliness of diagnosis and intervention [5, 7]. Current recommendations support endoscopic detorsion as the first‐line treatment in hemodynamically stable patients without signs of ischemia or perforation, while prompt surgical intervention is mandatory when conservative management fails or complications are suspected [2, 5, 7].

Herein, we report a case of SV causing acute intestinal obstruction in a 32‐year‐old woman at 27 weeks of gestation. This case highlights the diagnostic challenges, therapeutic decision‐making, and importance of multidisciplinary management in a rare but potentially life‐threatening condition during pregnancy.

## 2. Case Study

A 32‐year‐old woman, gravida 3 para 2, at 27 weeks + 0 days of gestation, was transferred by ambulance from a regional referral hospital (Gjilan, Kosova) to the obstetrics and gynecology clinic in Prishtina with a 3‐day history of progressive abdominal pain, abdominal distension, constipation, and vomiting. Her symptoms had worsened despite initial conservative management at the referring institution.

On admission, the patient was alert, oriented, and hemodynamically stable. Physical examination revealed a markedly distended abdomen with generalized tenderness and tympany on percussion. Bowel sounds were absent during auscultation. There were no signs of peritonitis. The patient was afebrile and clinically dehydrated, with a pulse rate of 88 beats/min and blood pressure of 120/80 mmHg. Chest examination was unremarkable.

A gynecological examination showed a normal vulva and vagina. The cervix was closed and of appropriate length for gestational age. Transabdominal obstetric ultrasound demonstrated a viable singleton pregnancy consistent with gestational age (27 weeks + 3 days), posterior placenta, normal amniotic fluid volume, and normal umbilical artery Doppler flow. No free fluid was detected in the peritoneum or Douglas pouch.

Initial laboratory analysis showed mild anemia, leukocytosis at the upper limit of normal, and an elevated C‐reactive protein (CRP) level (40.3 mg/L). Renal and liver function tests were within normal limits. Urinalysis revealed leukocyturia, while vaginal swab culture showed no pathogenic growth.

A gastroenterology consultation noted abdominal rigidity on palpation with resonant percussion. Digital rectal examination revealed an empty rectum. Placement of a rectal tube did not result in gas or fecal decompression. Given the suspicion of intestinal obstruction, abdominal imaging was pursued.

MRI of the abdomen demonstrated marked dilatation of the sigmoid colon with air–fluid levels and a transition point in the left abdomen, findings consistent with SV, without evidence of bowel ischemia or perforation (Figure 1). A small amount of perihepatic and perisplenic fluid was noted; solid organs were otherwise unremarkable.

Figure 1MRI of the sigmoid volvulus in pregnancy. The magnetic resonance imaging of the abdomen shows a dilated sigmoid volvulus in the left upper quadrant that has an upside‐down U‐shape, resembling a “coffee‐bean” appearance (A). The same patient’s sigmoid volvulus view in cross‐section (B). The figure also displays the fetus.

**Figure:**
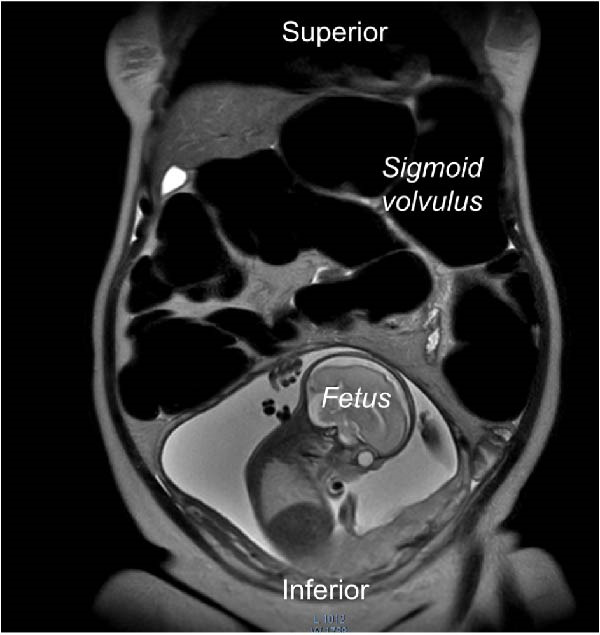
(A)

**Figure:**
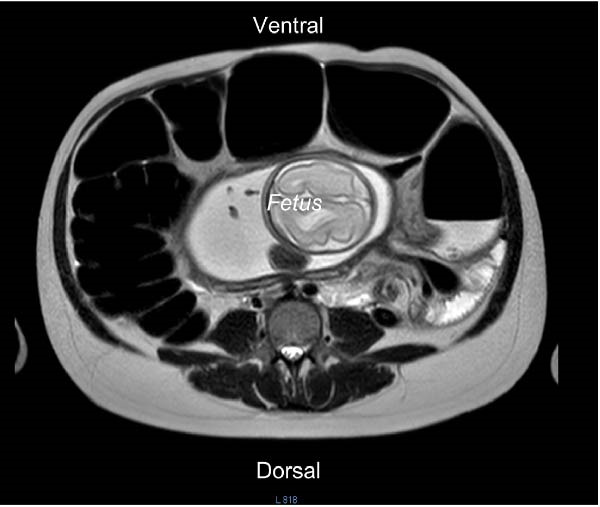
(B)

Flexible colonoscopy confirmed a torsion at the rectosigmoid junction. Endoscopic detorsion, the first‐line treatment in the absence of ischemia, was attempted but was unsuccessful. Following transient symptom relief, the patient experienced rapid recurrence of abdominal distension and pain. A second colonoscopic detorsion attempt also failed.

After multidisciplinary discussion involving general surgeons, obstetricians, anesthesiologists, and the patient, surgical intervention was recommended. Given the absence of fetal distress and extreme prematurity, continuation of pregnancy was prioritized.

The patient was transferred to the abdominal surgery clinic and underwent exploratory laparotomy under general endotracheal anesthesia via a midline incision. Intraoperatively, the gravid uterus was displaced cranially. A 360° counterclockwise volvulus of a markedly dilated sigmoid colon with significant mesenteric congestion was identified. No perforation was present, but bowel edema and compromised perfusion were evident.

**Figure 2:**
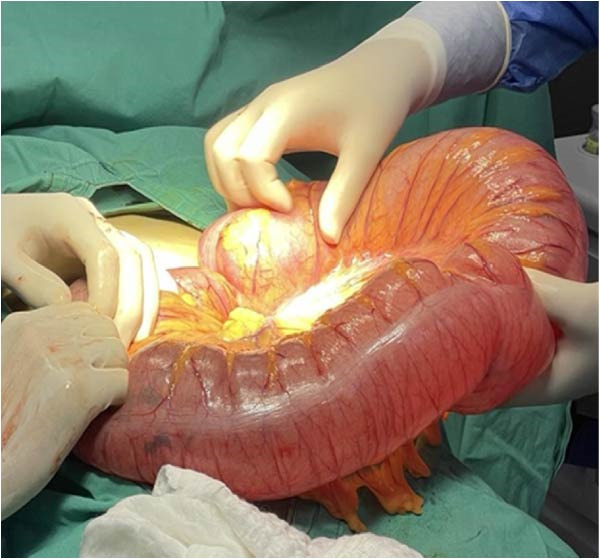
Sigmoid volvulus.

**Figure 3:**
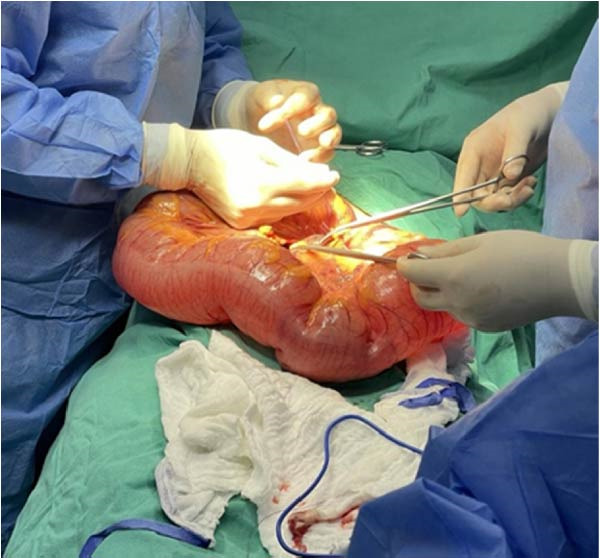
Sigmoid resection according to Hartman.

**Figure 4:**
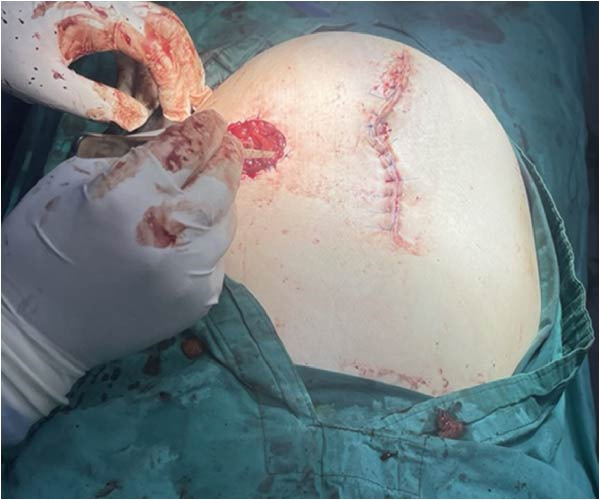
Colostomy.

The surgical team performed a sigmoid colectomy with end colostomy (Hartmann procedure) due to the bowel condition, increased intraabdominal pressure from the gravid uterus, and the high risk of anastomotic failure. The procedure was completed without intraoperative complications. Both maternal and fetal conditions remained stable throughout surgery (Figures 2, 3 and 4).

Postoperatively, the patient was monitored for 3 days. Fetal surveillance confirmed normal heart activity and appropriate growth. The patient received intravenous fluids, analgesics, prophylactic anticoagulation, antibiotics, proton pump inhibitors, and vitamin supplementation. Bowel function improved, and the colostomy was functional. She was discharged in good general condition.

At 38 weeks and + 1 day of gestation, the patient was readmitted with the colostomy in situ. Obstetric examination showed early labor with cephalic presentation. Given her surgical history, cesarean delivery was performed at 38 weeks + 4 days, resulting in the birth of a healthy neonate weighing 3040 g, with an Apgar score of 9/10. The postoperative course was uneventful, and the patient was discharged 4 days later with planned follow‐up by the abdominal surgery team for future intestinal continuity restoration.

## 3. Discussion

SV is an uncommon cause of large‐bowel obstruction that is more frequently encountered in elderly patients with chronic constipation or a redundant sigmoid colon [2, 8]. However, when it occurs during pregnancy, it represents the most common cause of colonic obstruction in pregnant women, particularly in the third trimester when the enlarging uterus alters bowel mobility and position [9]. According to the most recent systematic review by Augustin et al. [10], fewer than 200 cases have been reported worldwide, highlighting both the rarity of the condition and the diagnostic challenges it poses. In the last few years, we have not reported a single case in our clinic. Chronic constipation and pregnancy may be the causes of the longer sigmoid colon. During the growth of the uterus in pregnancy, the pressure in the sigmoid colon increases, causing its obstruction [6]. In our case, we admitted the patient to the obstetrics and gynecology clinic in Prishtina due to severe abdominal pain, vomiting, fatigue, and constipation that persisted for 10 days and worsened.

Several physiological and anatomical changes during pregnancy predispose to SV. Progesterone‐mediated smooth muscle relaxation results in reduced bowel motility, while the progressively enlarging uterus displaces and elongates the sigmoid colon, increasing the risk of torsion around its mesenteric axis. Chronic constipation, multiparity, and a redundant sigmoid colon further contribute to this risk [6, 9, 10]. These factors were present in our patient and likely played a role in disease development.

Clinical presentation of SV in pregnancy is often nonspecific and overlaps with common pregnancy‐related gastrointestinal symptoms, such as abdominal distension, nausea, vomiting, and constipation [4, 8, 10]. Importantly, symptoms themselves are not delayed; rather, diagnosis and definitive treatment are frequently postponed because complaints may initially be attributed to normal pregnancy changes. This diagnostic delay is a critical determinant of outcome [10], as prolonged obstruction may progress to bowel ischemia, necrosis, perforation, sepsis, and fetal compromise.

Maternal and fetal outcomes in SV depend primarily on bowel viability at the time of surgery. According to the literature [8, 10], maternal mortality rates for SV are ~5% in cases without bowel ischemia, rising dramatically when necrosis or perforation is present. Fetal mortality remains substantial, particularly when intervention is delayed or the interval between surgery and delivery is short.

Diagnostic imaging in pregnancy requires balancing diagnostic accuracy with fetal safety, especially by avoiding unnecessary fetal radiation exposure [2, 10]. While plain abdominal radiography, such as computed tomography (or CT), may demonstrate characteristic findings of SV, MRI is currently the preferred modality when available, as it avoids ionizing radiation and provides superior soft‐tissue contrast for detecting bowel dilatation, transition points, and mesenteric twisting [2, 8, 10]. Ultrasonography has limited utility for direct diagnosis but may raise suspicion by demonstrating bowel dilatation or free fluid. In our case, MRI findings were pivotal in confirming the diagnosis and guiding management, in accordance with current radiological recommendations for acute abdomen in pregnancy [2, 10].

Management of SV during pregnancy should follow a stepwise approach and be individualized based on gestational age, bowel viability, and maternal–fetal status [2]. Endoscopic detorsion is widely recommended as the first‐line treatment in stable patients without signs of ischemia or perforation, regardless of gestational age [6, 9, 10]. The successful colonoscopic detorsion was associated with reduced maternal and fetal mortality, supporting its role as an initial intervention when feasible [10]. However, recurrence rates after detorsion are high, and failure of endoscopic decompression—especially in the presence of ongoing distension, recurrent symptoms, or suspected ischemia—necessitates surgical intervention. In our patient, two attempts at colonoscopic detorsion were unsuccessful, mandating operative management. Definitive surgery typically involves resection of the affected segment. A Hartmann procedure (resection with end colostomy) may be preferred over primary anastomosis in the setting of bowel edema, congestion, and increased intraabdominal pressure from pregnancy, as this reduces the risk of anastomotic leakage.

Surgical strategy must consider both maternal safety and fetal well‐being [2, 8, 10]. Although cesarean delivery is technically feasible at 27 weeks’ gestation, it is associated with significant neonatal morbidity. In the absence of fetal distress, continuation of pregnancy is generally recommended [5, 10]. The decision to perform a Hartmann procedure in our case was based on marked bowel edema, mesenteric congestion, increased intraabdominal pressure from the gravid uterus, and the elevated risk of anastomotic failure. Deferring intestinal continuity restoration until after pregnancy is consistent with published recommendations and aims to minimize surgical risk to the mother.

This case illustrates the need for a multidisciplinary approach involving obstetricians, surgeons, anesthesiologists, radiologists, and gastroenterologists. Early collaboration facilitates timely diagnosis, appropriate imaging, and optimal therapeutic decision‐making, ultimately improving maternal and fetal outcomes.

## 4. Conclusion

SV during pregnancy is a rare but potentially life‐threatening cause of intestinal obstruction, with diagnosis often complicated by symptom overlap with normal gestational changes. Early clinical suspicion and appropriate imaging—preferably MRI—are essential to prevent diagnostic delay. Management should follow a stepwise approach, with endoscopic detorsion as first‐line treatment in stable patients without signs of ischemia and prompt surgical intervention reserved for failed decompression or compromised bowel viability. In certain instances, sigmoid resection with end colostomy is a secure and efficacious choice, facilitating the continuation of pregnancy and enhancing fetal maturity while reducing maternal risk.

## Author Contributions

Berat Krasniqi designed the study and performed the day‐by‐day experimental work and revision. Arben Haliti assisted in surgery and revision. Mentor Sopjani supervised the work, paper drafting, and revising. All authors reviewed the manuscript.

## Acknowledgments

The authors have nothing to report.

## Funding

This research received no specific grant from any funding agency in the public, commercial, or not‐for‐profit sectors.

## Disclosure

All authors have read and approved the final version of the manuscript. Berat Krasniqi had full access to all the data and took complete responsibility for the integrity and accuracy of the analysis and affirms that this manuscript is an honest, accurate, and transparent account of the study being reported.

## Consent

Appropriate informed consent was obtained from the patient for publication of this case report and accompanying images.

## Conflicts of Interest

The authors declare no conflicts of interest.

## Data Availability

The authors confirm that the data supporting the findings of this case report are available within the article.
